# Congenital heart defects genetic architecture in a small cohort: an integrated approach to prioritizing variants

**DOI:** 10.3389/fcvm.2026.1850208

**Published:** 2026-07-06

**Authors:** Anna V. Korobeinikova, Ekaterina S. Petriaikina, Dmitry I. Tychinin, Vladimir S. Yudin, Naida I. Bulaeva, Sayaly M. Gyulmamedova, Georgy A. Khugaev, Tatiana V. Sukhacheva, Ekaterina A. Snigir, Sergey I. Mitrofanov, Antonina M. Rumyantseva, Dmitry V. Svetlichnyy, Sergey M. Yudin, Elena Z. Golukhova, Veronika I. Skvortsova

**Affiliations:** 1 Federal State Budgetary Institution Centre for Strategic Planning and Management of Biomedical Health Risks of the Federal Medical and Biological Agency (Centre for Strategic Planning of the Federal Medical and Biological Agency), Moscow, Russia; 2 A.N. Bakulev National Medical Research Center for Cardiovascular Surgery of the Russian Ministry of Health, Moscow, Russia; 3 The Federal Medical Biological Agency (FMBA of Russia), Moscow, Russia

**Keywords:** CHD, children, congenital heart defects, pathogenic genetic variants, SNP, WGS, whole-genome sequencing

## Abstract

**Introduction:**

Congenital heart defects (CHD) constitute a prevalent group of structural birth anomalies, characterised by substantial genetic heterogeneity and diverse clinical phenotypes.

**Methods:**

To investigate the underlying genetic architecture, we performed whole-genome sequencing (WGS) in a cohort of 50 patients with echocardiographically confirmed CHD, followed by systematic variant identification and functional annotation.

**Results:**

Our analysis reveals the limited discriminatory capacity of current genomic annotation databases and underscores the necessity of stratifying genetic risk assessments by specific CHD subtypes. By integrating clinical classifications, genomic data, and tissue-specific expression profiles, we identified novel coding and non-coding variants alongside putative regulatory signals that may contribute to CHD pathogenesis. Within cardiac-specific genes, we identified CHD subtype-specific genetic associations, including JARID2 with PDA, GOSR2/TBX18 with VSD, PCDHA9 with ASD, and a multi-gene signature (CREBBP, ZFPM2, SLC27A6, ADAM17, ETS1) with atrioventricular septal defects. Among coding variants in non-CHD-associated genes, we identified COL11A2 and PCOLCE2 as plausible collagen-related candidates for CHD pathogenesis.

**Discussion:**

These findings reinforce the polygenic architecture of CHD and highlight the value of context-aware, phenotype-driven interpretation of genetic variants. Collectively, this study expands the understanding of the genetic landscape underlying congenital heart anomalies and emphasises the need for larger, deeply phenotyped cohorts to translate these preliminary insights into clinically applicable predictors.

## 1 Introduction

Congenital heart defects (CHD) represent one of the most common forms of congenital anomalies, affecting approximately 1% of newborns worldwide. These structural abnormalities of the cardiovascular system, arising during intrauterine development, include various defects such as malformations of the heart chambers, valves, and major vessels, and are among the leading causes of morbidity and mortality in infants [1]. In 2022, around 14,000 children were born with CHD in Russia, of whom 60%–70% required surgical intervention within the first year of life [2]. Despite a slight decrease in the proportion of CHD in infant mortality to 42.7%, the issue remains relevant and demands both improvements in therapeutic and surgical correction methods and the development of preventive approaches to reduce the risk of CHD [2]. Understanding the genetic factors underlying the formation of congenital heart anomalies will help reduce the risk of developing CHD during pregnancy planning, making this area of research highly relevant today.

The development of CHD is driven by complex interactions between genetic and environmental factors. Modern research in this area focuses on identifying biomarkers for early detection of predisposition to these anomalies [3, 4], which is crucial for prognosis and outcome prediction. The genetic basis of CHD includes chromosomal abnormalities, deletions, duplications, and point mutations. Monogenic forms account for only a small portion of cases, while the remaining cases are believed to involve multiple rare variants interacting with each other and environmental factors [5, 6]. Despite the known role of certain genes involved in cardiovascular development, most cases of CHD remain without an identified genetic cause, highlighting the need for further studies to understand the mechanisms underlying these anomalies, identify their key genetic factors, and assess their clinical significance [7].

Association studies, such as Genome-Wide Association Studies (GWAS), are currently a key method for identifying genetic variants linked to diseases. This approach allows analysis of millions of single nucleotide polymorphisms (SNPs) across the genome, detecting statistically significant correlations between genetic markers and clinical phenotypes. Over the past two decades, GWAS has identified hundreds of loci associated with the risk of developing a wide range of conditions. The data obtained have laid the foundation for personalized medicine, where genetic markers are used to predict risks, optimize therapy, and develop targeted biomarkers [8]. However, despite these successes, the GWAS methodology faces fundamental limitations that call into question its clinical applicability, especially when dealing with rare diseases or unique populations [9].

Traditional GWAS approaches in CHD face the same challenges as in other rare diseases: limited sample sizes, high genetic heterogeneity, and the difficulty of translating associations into causality [10]. In cases where sample sizes are small and traditional GWAS approaches lose statistical power, this work implements an alternative approach that combines several stringent filters aimed at identifying functionally significant genetic signals. The methodology is based on analyzing rare variants (allele frequency in the gnomAD database <0.1%), with pathogenicity assessed by a CADD score >20 and minimal prevalence in the studied cohort (>15%). Additionally, association analysis was performed using Fisher’s exact test on pre-selected genes linked to CHD development according to literature data.

## 2 Materials and methods

### 2.1 Experimental design and preparation of biomaterials

The study cohort included 50 patients with CHD recruited at the Federal State Budgetary Institution “A.N. Bakulev National Medical Research Center for Cardiovascular Surgery” from 2019 to 2021. Demographic and clinical data were collected retrospectively from medical records and electronic registries. Inclusion criteria: a confirmed diagnosis of congenital heart disease (CHD) according to ICD-10 (codes Q21–Q25) in accordance with the current specialized clinical guidelines of the European Society of Cardiology (https://scardio.ru/rekomendacii/rekomendacii_esc) or the Russian Society of Cardiology (https://scardio.ru/rekomendacii/rekomendacii_rko), age 0–18 years. The presence of a structural cardiac anomaly was confirmed by echocardiography according to standard imaging protocols. Exclusion criteria: concomitant neonatal pathologies not associated with CHD (e.g., congenital infections, severe metabolic disorders), as well as a history of carriage of dangerous infections: human immunodeficiency virus, hepatitis B, hepatitis C, and syphilis. The presence of congenital heart defects in the context of genetic syndromes was not an exclusion criterion. A peripheral blood or cardiac tissue sample was obtained from each participant, followed by whole-genome sequencing. The control group—100 people—was formed from conditionally healthy participants of the Database of Population Frequencies of Genetic Variants of the Population of the Russian Federation of the Federal State Budgetary Institution “Center for Clinical and Practical Research” of the Federal Medical and Biological Agency of Russia (https://gdbpop.nir.cspfmba.ru/). Individuals in the control group were included only in the absence of documented personal or familial history of congenital heart defects or other major congenital anomalies (Supplementary Material).

### 2.2 DNA extraction, sequencing library preparation, and WGS of blood samples

Genomic DNA was extracted manually from whole blood and tissue samples using the QIAamp DNA Mini Kit (Qiagen, Germany). Tissue homogenization was performed in 80 μL of sodium phosphate buffer using the Bioprep-24 Homogenizer (Allsheng, China). The DNA elution was carried out in 100 μL of AE buffer. The concentration of the extracted DNA was determined using the QuantiFluor ONE dsDNA assay kit (Promega) on a Quantus fluorometer (Promega, USA). DNA quality was assessed using NanoDrop 8,000 (Thermo Fisher Scientific, USA).

Sample preparation of whole-genome libraries was performed using Illumina DNA Prep reagent kit according to the manufacturer’s recommendations (Document $N^{o}$ 1000000025416 v10, Illumina, USA) and IDT-ILMN Nextera DNA UD Indexes Set A and Set B to prevent cross-contamination of samples. The concentration of the libraries was measured on an Infinite F Nano Plus plate reader. The size of the resulting libraries was determined using the Agilent D1000 reagent kit on an Agilent 4,200 TapeStation (Agilent Technologies, USA). Pooling was performed automatically using a Tecan Freedom EVO robotic station (Tecan, Switzerland). Pool quality control was performed using Agilent HS D1000 ScreenTape reagent kit an Agilent 4,200 TapeStation (Agilent Technologies, USA). Whole-genome sequencing was performed on an Illumina NovaSeq 6000 instrument using the S4 reagent kit for 300 cycles (Illumina, USA) for paired-end reads of 2 × 150 bp.

### 2.3 Bioinformatic processing and calling of small genetic variants

Bioinformatics analyses were conducted using a uniform computational workflow applied consistently across both study cohorts. For reads in the FASTQ.GZ format, quality control was performed using the bioinformatics tool FastQC v0.11.9 (08.01.2020) (https://www.bioinformatics.babraham.ac.uk/projects/fastqc/). Then, the reads were aligned to the reference genome using the Illumina DRAGEN Bio-IT Platform v07.021.510.3.5.7 (17.02.2020), rev. 1.0 (17.02.2020) (https://www.illumina.com/science/genomics-research/articles/popgen-variant-calling-with-dragen.html). The genomic sequence GRCh38.d1.vd1 (https://gdc.cancer.gov/about-data/gdc-data-processing/gdc-reference-files) was used as the reference sequence. Quality control of the alignment results (in BAM format) was performed using the internal algorithms of the DRAGEN platform and additional tools [samtools v1.13 [11], Qualimap [12], mosdepth v0.3.1 [13]]. The average genome coverage for the analyzed samples was at least 30X, and the percentage of reads mapping to the genome was over 98%. Comparable sequencing quality metrics were observed across all study samples (Supplementary Table S1).

Minor genetic variants (single-nucleotide variants, as well as insertions and deletions up to 50 bp) were detected using Manta v1.6.0 (09.07.2019) [14] and Illumina Strelka2 v2.9.10 (08.11.2018) [15] with default settings. The resulting minor variants were normalized using bcftools norm v1.16 (18.08.2022) [11]. The number of minor variants in VCF files was estimated using bcftools stats v1.15 (21.02.2022) [11]. Only variants with a call quality score greater than 30 (QUAL > 30) were retained for further analysis, and filtering was applied to include only “PASS” variants, excluding those flagged with low quality annotations.

The filtered VCF files were converted to PLINK binary format (BED/BIM/FAM) using PLINK v2.0 (–make-bed) [16, 17]. A rigorous quality control filtering procedure was then applied to the variant call set using PLINK v2.0 with the following thresholds: exclusion of variants deviating from Hardy-Weinberg equilibrium (HWE *p*-value <1 × 10, −hwe $1\times 10^{-6}$), removal of variants with minor allele frequency below 5% (–maf 0.05), exclusion of samples with more than 1% missing genotypes (–mind 0.01), and removal of variants with genotype missingness exceeding 1% (–geno 0.01).

The present analysis did not include structural variants (SVs) or copy number variants (CNVs). The modest sample size precluded adequate statistical power for robust SV/CNV detection, quality assessment, and aggregate burden testing. Additionally, the computational workflow was intentionally designed for single nucleotide polymorphism (SNP) association mapping.

### 2.4 Clinical interpretation of variants in the ClinVar database

For clinical interpretation of genetic variants, the OpenCRAVAT tool with the ClinVar module (2023-10 release) was used. Database queries were executed using a keyword-based search strategy with the following terms connected by Boolean OR operators: heart | cardiac | cardiomyopathy | congenital | septal | tetralogy | fallot | transposition | hypoplastic | coarctation | aortic | pulmonary | tricuspid | mitral | ventricular | atrial | patent | ductus | heterotaxy | situs | conotruncal | noonan | leopard | williams | alagille | holt-oram | char | diGeorge | velocardiofacial | 22q11. Variants were considered clinically significant if they were classified in the ClinVar database as Pathogenic, Likely pathogenic, or Pathogenic/Likely pathogenic.

### 2.5 Cohort-enriched rare variants detection

To identify cohort-enriched rare variants characteristic of a group of patients with congenital heart defects, a combined approach combining bootstrap sampling, rare variant analysis, and statistical analysis was used. In the first stage, stochastic sampling of the cohort (30 individuals in each group) was performed, after which variants meeting the following criteria were selected: complete absence in the control subgroup (AF = 0%) and an occurrence in the patient subgroup of more than 25%. A total of 100 iterations were performed, and variants detected in more than 90 iterations were included in the preliminary list of candidates (multiple iterations and a threshold of 90% of detections minimize stochastic noise). In the second stage, statistical validation was performed using the Fisher exact test in PLINK v2.0, which took into account the full sample. Correction for multiple testing was performed using the Benjamini-Hochberg method. In the third stage, rare variants were analyzed using two approaches: individual selection of rare variants (GnomAD AF < 0.1%) with high pathogenic potential (CADD > 20, DANN > 0.5) and a Burden test to assess the enrichment of rare variants in genes clinically significant for cardiac diseases (adjusted for gene length and multiple testing). Mutation burden was calculated as the total number of detected mutations in the coding region of DNA divided by the size of the region under study. Final selection required compliance with strict quality criteria: coverage depth > 20x, call quality > 30.

### 2.6 Functional annotation

To assess the regulatory activity of non-coding SNPs, RegulomeDB was used, considering categories 1a-2b, which indicate experimentally confirmed effects on transcription factor binding or regulatory activity. The association between phenotypes and variants was strengthened by incorporating eQTL data (Gtex v.10), focusing on data from the heart and aorta.

### 2.7 Single-cell sequencing data analysis

To assess gene expression of individual cell populations, a publicly available cell atlas (https://www.heartcellatlas.org) was used; a processed AnnData object [H5AD (log-normalized)] with cell annotation from the authors was loaded. Gene expression visualizations were performed using the scanpy package (1.9.5). Expression modules were assessed using the built-in function sc.tl.score_genes with standard parameters, except for the flag ctrl_size = 10; due to the small number of genes in the analyzed signatures.

## 3 Results

### 3.1 Sample structure and study design

**Figure 1:**
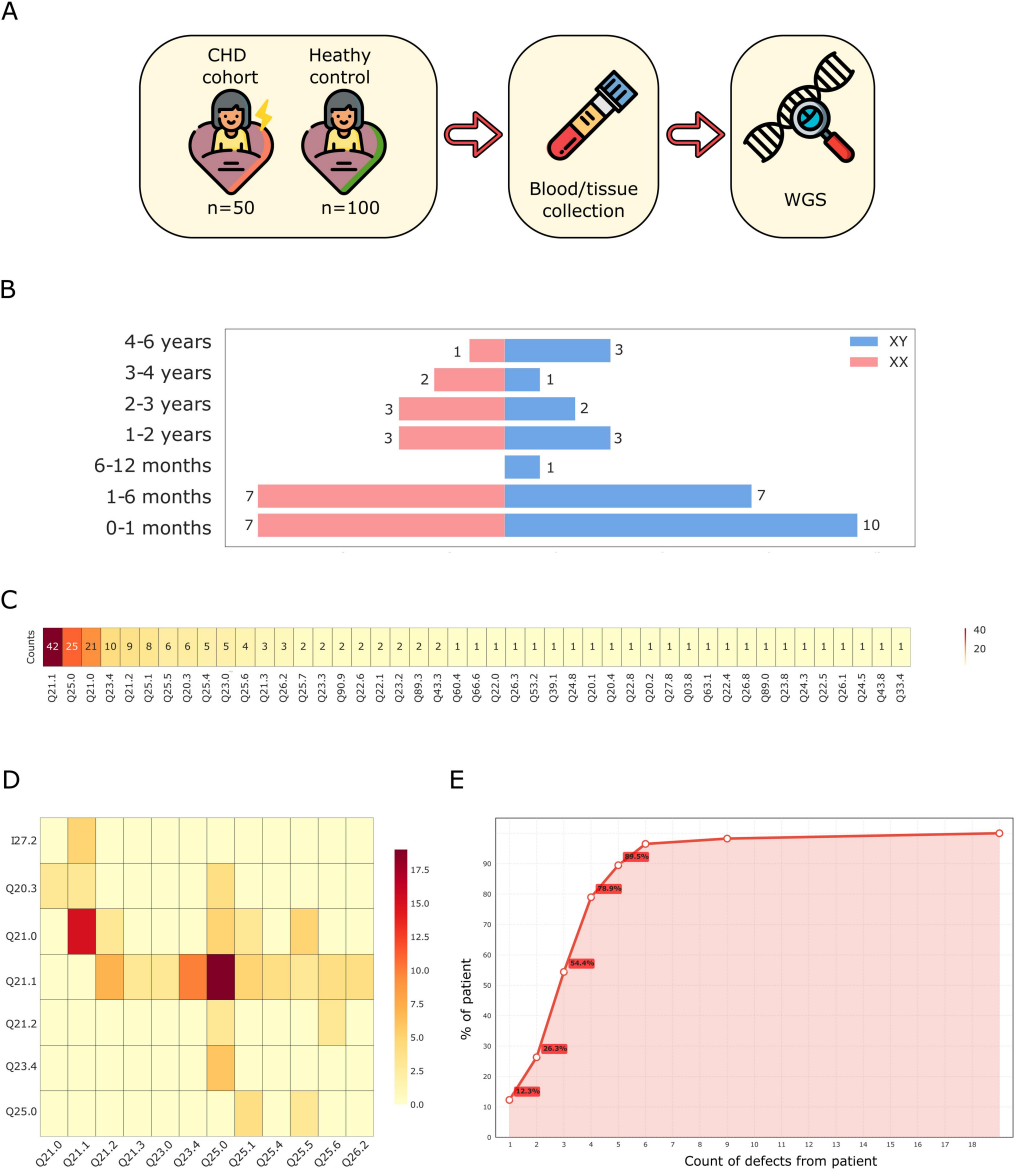
Key demographic and clinical characteristics of the sample **(A)** Samples and study design. **(B)** Age and sex distribution of the CHD cohort. **(C)** Distribution of congenital heart defects in the study sample. **(D)** Combination of CHD within the cohort. **(E)** Presence of multiple CHD within the cohort.

To assess the contribution of congenital genetic factors to the development of structural cardiac anomalies, a sample of 50 children with congenital heart defects (Figure 1A, Supplementary Material) and a control group of 100 participants without CHD were formed. A blood or cardiac tissue sample was obtained from each participant, followed by DNA extraction and whole-genome sequencing (Figure 1A). The sample was characterized by an even gender distribution (54% boys, 46% girls) with a significant predominance of infants (0–1 year–64%, *n* = 32), which corresponds to the typical age of CHD manifestation (Figure 1B). The control sample was represented by adult participants with a comparable gender composition (Supplementary Figure S1). Within the sample, the majority of patients had combined heart defects, including classic congenital heart defect combinations (Q21.3: tetralogy of Fallot, etc.), as well as rarer combinations of valve and chamber structural anomalies (Supplementary Figure S2). The most frequently recorded defects within the cohort under consideration were Q21.0: ventricular septal defect (VSD), Q21.1: atrial septal defect (ASD), Q25.0: patent ductus arteriosus, Q21.2: atrioventricular septal defect (AVSD) (Figure 1C); these defects were also frequently recorded in combination in a single patient (Figure 1D). It is worth noting that within the sample, there are participants with a combination of congenital heart defects and non-cardiac structural anomalies; however, these combinations are represented by isolated observations (Figure 1C). More than half of the sample participants have 3 or more combined congenital defects (Figure 1E).

### 3.2 Annotation of variants by the ClinVar clinical database

**Figure 2:**
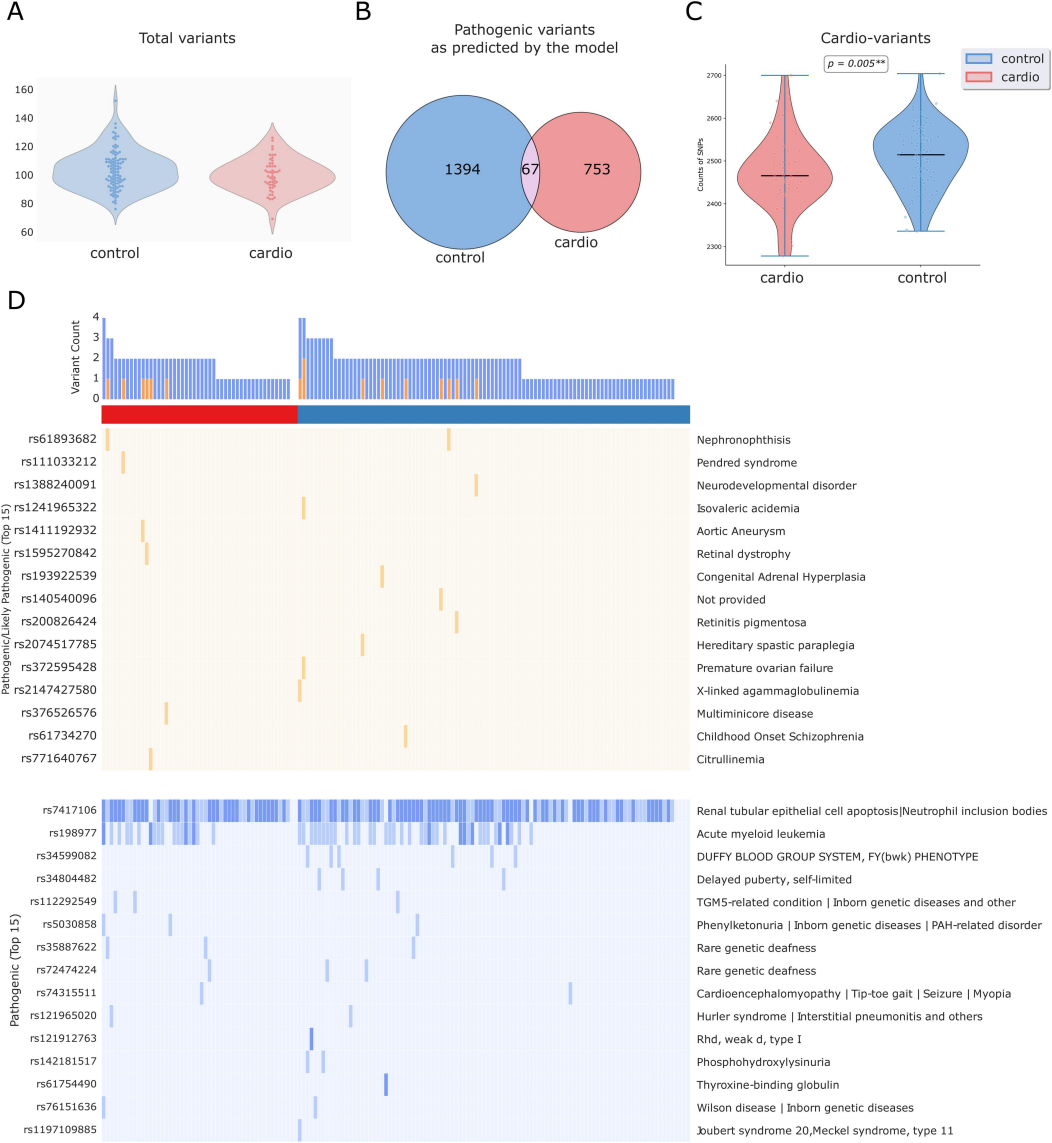
Annotation of variants by the ClinVar clinical database **(A)** Distribution of the number of pathogenic and likely pathogenic variants per sample. **(B)** Intersection of pathogenic/likely pathogenic variants between groups. **(C)** Distribution of ClinVar cardiac SNPs across cohorts. **(D)** Heat map of the most common pathogenic/likely pathogenic variants with the annotation provided.

As part of the initial assessment of the contribution of inherited genetic variants, the frequency of pathogenic and likely pathogenic variants annotated in the ClinVar database was analyzed in the context of the studied cardiac pathology (Figure 2). The distribution of the number of such variants per sample (Figure 2A) demonstrates the absence of statistically significant differences between the patient and control groups (patient group: 99.76 ± 11.5; control: 103.46 ± 13.66; Mann-Whitney test, *p* = 0.152). This suggests the absence of systematic accumulation of clinically known pathogenic alleles in the studied cohort. Limited overlap of pathogenic and likely pathogenic variants was also observed between the groups, suggesting genetic heterogeneity of the compared cohorts and the potential specificity of the considered SNPs for differentiating the cohort of interest (Figure 2B). Stratified analysis of cardiac-specific ClinVar SNPs demonstrated a statistically significant enrichment of these variants within the control cohort (Figure 2C). Subsequent analyses revealed no cohort-specific differences in genotype distributions (homozygous vs. heterozygous; Supplementary Figure S3A) or in the frequency of pathogenic variant calls (Supplementary Figure S3B). The overrepresentation of likely benign SNPs with multiple independent submissions in the cardiac cohort highlights potential limitations in current functional annotation pipelines and warrants systematic re-evaluation (Supplementary Figure S3C). To refine variant prioritization, we interrogated the overlap between ClinVar pathogenic/likely pathogenic SNPs and variants mapped to CHD-associated genes in curated public resources (https://chdgene.victorchang.edu.au; https://chddb.fwgenetics.org); Supplementary Figure S3D). Distributional analysis of variants retained after the aforementioned filtering steps confirmed the absence of cohort-specific biases in variant representation (Supplementary Figure S3E). Analysis of the heatmap displaying the top 15 most frequently encountered pathogenic variants in the studied cohort also demonstrates a lack of clear signals (Figure 2D). The detected variants were annotated in ClinVar as associated with non-cardiac pathologies (e.g., neuromuscular diseases), indicating a rather random distribution of the SNPs in question across the samples and confirming the limited applicability of the existing annotations for cardiogenetic studies.

### 3.3 Investigation of variants in CHD-related genes

**Figure 3:**
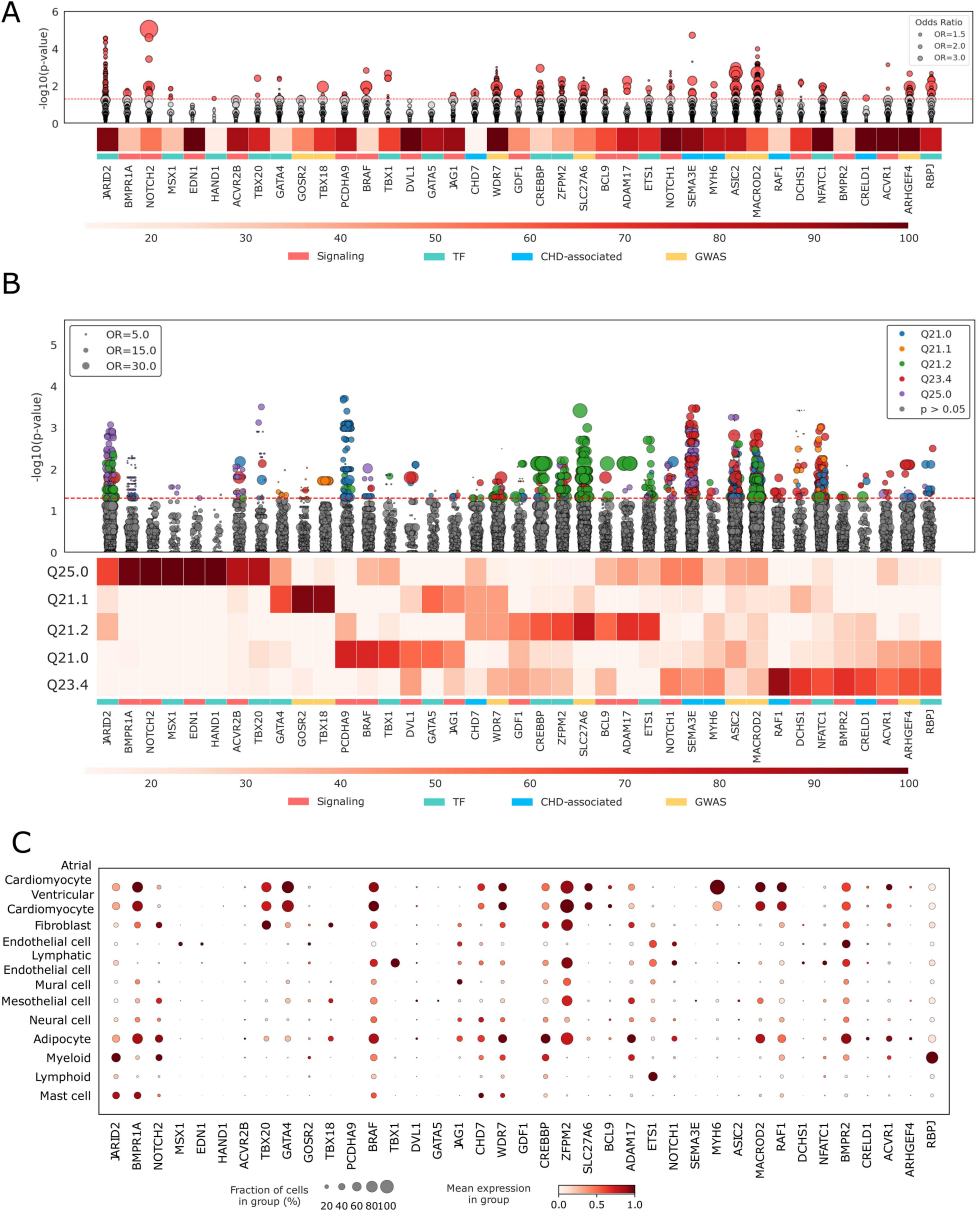
Mutation burden analysis in known CHD-associated genes **(A)** Mutation burden of the full cohort in functional categories of genes associated with CHD (the heat map visualizes mutation burden profiles, where the color gradient corresponds to the proportion of patients with variants in each gene), with the assessment of between-group variation in variants using Fisher’s exact test with Benjamini–Hochberg multiple comparison correction (FDR < 0.05). **(B)** Mutation burden in CHD subgroups in functional categories of genes associated with CHD (the heat map visualizes mutation burden profiles, where the color gradient corresponds to the proportion of patients with variants in each gene), with the assessment of between-group variation in variants using Fisher’s exact test with Benjamini–Hochberg multiple comparison correction (FDR < 0.05). **(C)** Expression of key genes characterized as specific for CHD in cell types of the cardiac atlas (https://www.heartcellatlas.org/).

Using an alternative method for identifying congenital heart defects-associated SNPs in the study cohort, a list of 39 genes associated with congenital heart defects was generated based on literature data [18–20] and public CHD-base data (https://chdgene.victorchang.edu.au; https://chddb.fwgenetics.or, Supplementary Table S2. The genes were classified into three functional categories reflecting their biological significance: signaling pathway genes (red), transcription regulators (green), and genes for syndromic forms of congenital heart defects (blue) (Figures 3A,B). Genes that showed association with congenital heart defects in a GWAS analysis [10] are shown in a separate block (yellow).

Analysis of the mutational burden of the complete sample in the formed gene groups revealed heterogeneity in the representation of polymorphisms between genes (Figure 3A, Supplementary Table S3). Thus, for 8 genes (END1, DVL1, WDR7, NOTCH1, NFATC1, CRELD1, ACVR1, ARHGEF4), at least one sequence variant (regardless of predicted pathogenicity) was identified in 100% of the analyzed patients, indicating that these genes are nearly universally altered at the DNA sequence level in our CHD cohort. For an integrated assessment of the total mutational burden for all 39 genes, the Total Burden Score was calculated—the sum of the number of heterozygous and homozygous variants per patient. Comparison between the patient group and the control sample demonstrated a statistically significant increase in the burden in the study cohort (*p* = $4.83\times 10^{-2}$, Mann–Whitney *U* test; Supplementary Figure S4). Most identified variants were likely benign and non-coding (Supplementary Figure S5). Evaluation of the between-group variation using Fisher’s exact test (Figure 3A) revealed the highest significance for the following genes: *JARID2* (chr6_15493658_A/C, *p* = 1.02–07, OR = 8.17), *NOTCH2* (chr1_120023570_T/G, *p* = $9\times 10^{-6}$, OR = 43.2), and *SEMA3E* (chr7_83617638_A/G, *p* = $3.3\times 10^{-5}$, OR = 5.67). Both genes are known to play a role in regulating embryonic cardiovascular development. *JARID2* is a key component of the Polycomb Repressive Complex 2 (PRC2), which is involved in the epigenetic control of gene expression important for cardiogenesis [21]. *NOTCH2*, in turn, plays a central role in the Notch signaling pathway, which is critical for cellular communication and differentiation in the developing heart [22]. *SEMA3E* is also one of the regulators involved in embryonic development; an association between mutations in this protein and the development of CHARGE syndrome, one component of which is cardiac abnormalities, has also been shown [23].

To further investigate the relationship between genetic variants and specific clinical forms of congenital heart disease, a similar analysis was conducted in groups of carriers of specific congenital heart disease (CHD) that were most represented in the study cohort (Q21.0, Q21.1, Q25.0, Q23.4, Q21.2). Calculation of the mutational load revealed an association between certain genes and congenital heart disease types and allowed for clustering within the gene pool under consideration (criterion: >50% representation of variants in the subgroup), while the use of Fisher’s exact test allowed us to identify the most significant polymorphisms (Figure 3B). Thus, the *JARID2* gene, previously identified in the overall cohort, showed the highest association with PDA (Q25.0), while the *GOSR2* and *TBX18* genes were associated with VSD (Q21.1), the *PCDHA9* gene was associated with the presence of ASD (Q21.0), and the *CREBBP, ZFPM2, SLC27A6, ADAM17*, and *ETS1* genes were associated with atrioventricular septal defects (Q21.2). Many genes showed cross-subtype associations, potentially reflecting both biological pleiotropy and phenotypic overlap within our cohort. For example, for the *SEMA3E* gene, a relationship was shown with both the carriage of left-sided hypoplastic heart syndrome (Q23.4) (chr7_83438120_G/T, *p* = 0.000348, OR = 7.43) and the presence of PDA (Q25.0) (chr7_83544616_A/G, *p* = 0.00093, OR = 5.24).

To test the reproducibility of GWAS signals associated with congenital heart defects, we analyzed SNPs previously identified by Lahm et al. [10] in association with various forms of CHD. In the study cohort, none of these polymorphisms showed statistically significant association (p>0.05, Fisher’s exact test) (Supplementary Table S4). For a number of variants (rs185531658, rs149890280, rs150246290, rs146300195, rs117527287), the odds ratio (OR) was zero, indicating the absence of these alleles in the sample. For the remaining SNPs, OR values ranged from 0.45 to 2.33, but all *p*-values exceeded the 0.05 threshold. Given the lack of significant associations at the level of the entire CHD group, further subgroup analysis appears uninformative. This lack of replication may be due to the limited size of our cohort, differences in the genetic structure of the populations, or heterogeneity of clinical phenotypes. However, in the genes studied (e.g., *SLC27A6, ARHGEF4, WDR7, GOSR2, TBX18*), we were able to identify other significant mutations in our cohort (Figure 3), supporting their potential role in the pathogenesis of CHD, albeit at the level of rare variants not identified in GWAS.

To assess potential pathogenicity, CADD and DANN scores were assigned to all variants (Supplementary Table S3). Fifty one SNPs across 17 genes (*ACVR2B, ARHGEF4, ASIC2, CREBBP, DCHS1, ETS1, GDF1, JARID2, MACROD2, PCDHA9, RBPJ, SEMA3E, SLC27A6, TBX1, TBX20, WDR7, ZFPM2*) were predicted as pathogenic by both tools (Supplementary Table S5). Variants in *ARHGEF4, CREBBP, DCHS1, GDF1, MACROD2, PCDHA9* and *SLC27A6* with DANN > 0.9 represent high-priority candidates for functional follow-up.

To assess the biological relevance of the identified associations, we used a public scRNA-seq atlas of the human heart. The atlas includes data on 12 annotated cell populations (Supplementary Figure S6A). Analysis of the expression profiles of the identified genes showed that some of them have clearly cell-specific expression patterns (Figure 3C). For example, the genes *CREBBP, ZFPM2*, and *SLC27A6*, previously shown to be associated with AVSD, demonstrate prominent expression in atrial and ventricular cardiomyocytes, endothelial cells, and adipocytes. However, not all examined genes are specifically expressed in cardiomyocytes. For instance, the genes *JARID2* and *ADAM17* exhibit the highest expression in blood immune cells, while the gene *PCDHA9*, associated with VSD, shows no expression within the analyzed cell types, which may be related to its pathogenic action occurring at early stages of embryonic development. A comprehensive analysis of the expression of all genes in the cluster (Supplementary Figure S6B) confirms the predominant expression of these genes in cardiomyocytes for carriers of VSD, ASD, PDA, and left heart hypoplasia, and in endothelial cells for DMPV, corresponding to the affected heart structures.

Thus, despite the limited overlap within specific SNPs, the evaluation of the mutation burden of the genes associated with CHD described in the literature, overall, within this small cohort, confirms their involvement in the pathogenesis of CHD. The specificity for developing certain CHD types is not observed for all genes, indicating that their pathogenic effects manifest at different stages of embryonic development.

### 3.4 Investigation of variants in other genes

**Figure 4:**
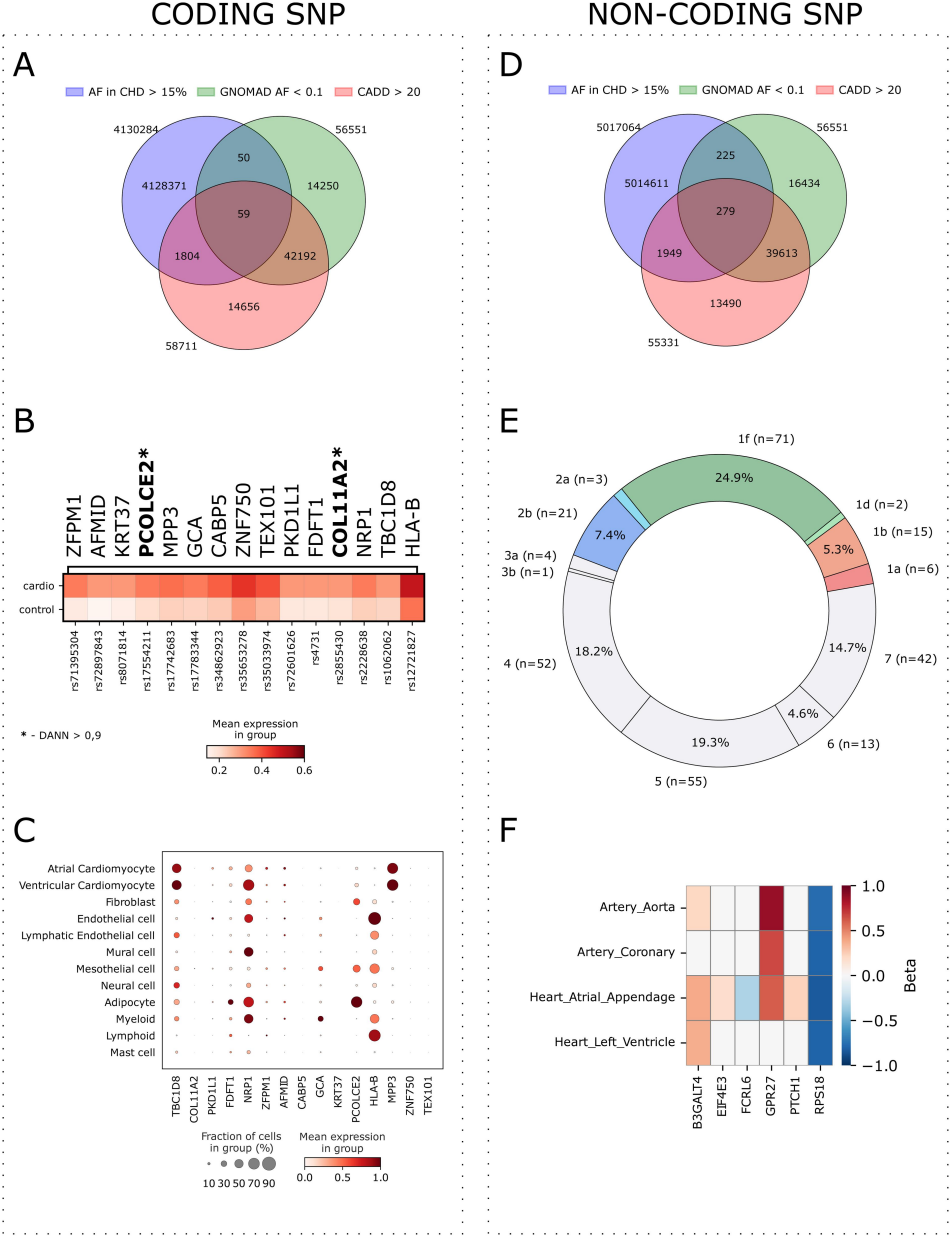
Cohort-enriched rare variant selection in other genes **(A)** Selection of mutations in the coding region. **(B)** Top 15 variants by LFC. **(C)** Evaluation of gene expression with coding CHD-associated SNPs found in the cell types of the cardiac atlas. **(D)** Selection of variants in the non-coding region. **(E)** Functional annotation using RegulomeDB. **(F)** Intersection with eQTL associations from GTEx v10.

To identify CHD-variants in other genes with risk associations with the development of CHD, a multi-tiered approach was used, combining stringent filters based on allele frequencies, functional manifestations, and prevalence in a fixed cohort. The selection criteria were as follows: allele frequency in gnomAD < 0.1, pathogenicity in CADD > 20, DANN > 0,5 (Supplementary Table S6) and prevalence in the patient cohort >15% (to identify the most representative signals). Data filters were applied to select 59 variants of genomic coding regions (Figure 4A, Supplementary Table S6), which were assigned to genes and protocols for predicting functional and statistical analysis. Among the top 15 genes identified new CHD SNPs, ranked by logarithmic change in allelic frequency in the patient cohort (Figure 4B), *TEX101*, CABP5, and *GCA* are particularly noteworthy, demonstrating AF > 0.5 for the identified SNPs in the CHD group. Additional DANN-based prioritization identified variants in two collagen-related genes, *COL11A2* and *PCOLCE2*, with prediction scores > 0.9 (Supplementary Table S7). Previous studies have implicated these genes in disorders associated with collagen dysfunction [24–26]. Given the established role of collagen biology in cardiac development [27], these findings suggest a potential contribution of the identified variants to CHD pathogenesis. Gene expression analysis revealed genes with potential for expression in atrial and ventricular cardiomyocytes, as well as endothelial cells, based on single-cell expression data (Figure 4C) for *TBC1D8, NRP1*, and *MPP3*, making these genes particularly interesting for future studies. Given the small cohort size (*n* = 50), the identified signals require validation to exclude sequencing artifacts and population stratification. Similar filters were applied to non-coding variants, revealing 286 signal non-coding variants (Figure 4D), 43% of which belonged to RegulomeDB categories > 2b (Figure 4E), with 7 variants exhibiting DANN scores > 0,9. This suggests a potential role in regulating gene expression, including tissue-specific decadal expression. To enhance biological interpretability, these signals were annotated with eQTL (expression quantitative trait loci) data from cardiac and aortic tissues taken from the GTEx v.8 project (Figure 4F, Supplementary Table S8). This allowed us to retain genes controlled by the observed variants. For example, *RPS18*, a gene encoding a component of a small number of ribosomal subunits, is downregulated in all analyzed cardiovascular diseases in carriers of the corresponding variants. This may indicate a systemic effect on the translational apparatus of cardiomyocytes. *GPR27*, a gene associated with neuroendocrine signaling, showed increased expression in carriers of a specific non-coding signal, exclusively in aortic tissues and, to a lesser extent, in coronary arteries and atria (atrial appendages). This tissue specificity suggests a possible involvement of *GPR27* in the regulation of vascular tone or remodeling.

Thus, the obtained data reveal unique new cohort-enriched rare unique variants variants in both coding and regulatory regions of the genome that may be associated with the development of congenital heart disease. The discovery of genes not previously associated with cardiogenesis opens the possibility of expanding existing knowledge about the molecular mechanisms of heart defect formation.

## 4 Discussion

The CHD formation is considered a pathological process with a multifactorial etiology [28]. Along with embryogenetic and environmental factors, the influence of genetic and epigenetic factors has been demonstrated as significant in the development of CHD [29, 30]. This study presents a comprehensive analysis of the potential application of accumulated knowledge about the genetics of CHD in a small cohort of patients. Both the prevalence of known pathogenic variants and the search for new variants within cardio-associated genes, as well as pathogenic variants in other genes, were analyzed. This study has several limitations. The modest sample sizes of the comparison cohorts, combined with the phenotypic complexity of the cardiac anomalies in most patients and the absence of karyotype data, precluded stratification into syndromic and non-syndromic CHD subgroups and restricted association analyses to the complex CHD rather than isolated CHD cases. Methodologically, pathogenic variants were identified solely through computational calling from WGS data without orthogonal validation by Sanger sequencing, and functional annotation relied on publicly available cellular atlases rather than experimental assays. Furthermore, the unavailability of parental biospecimens precluded the assessment of inheritance patterns (de novo vs. inherited) for the identified associated variants. Collectively, these constraints underscore the need for larger, deeply phenotyped cohorts with trio-based sequencing and orthogonal validation to confirm and extend our findings. The lack of data on the ethnic origin of the participants makes it impossible to assess the impact of origin on the reproducibility of the results. One of the key conclusions was the limitation of relying solely on annotations from publicly available databases, such as ClinVar. Despite their widespread use in clinical interpretation, we demonstrated that these resources are insufficiently specific in the context of cardiogenetics (Figure 2). This underscores the need to expand functional validation and to develop specialized reference databases for cardiovascular diseases.

GWAS enable the identification of key genetic loci associated with the risk of CHD. However, experience with GWAS within the CHD cohort did not reveal multiple genetic associations. For example, a recent study [10] identified only one significant SNP on chromosome 5q22.2, associated with all phenotypes of CHD and septal defects. This limitation of the results may be explained by combining all CHD variants into a single comparison group, which can dilute signals specific to individual CHD subtypes. For instance, in the analysis of patients with transposition of the great arteries, four significant SNPs were found in the *MACROD2* locus on chromosome 20p12.1. It was also shown that risk variants in the *GOSR2* locus on chromosome 17q21.32 are associated with anomalies of the thoracic arteries and veins. The absence of statistically significant associations for the variants described herein may be attributable to limited statistical power in our cohort, compounded by its relatively small sample size and considerable ethnic and phenotypic heterogeneity. Nevertheless, in our cardio-cohort, we identified the presence of potentially pathogenic SNPs within the genes associated with CHD, as presented in this study (Figure 3), suggesting the potential applicability of analyzing variations in this set of genes for CHD diagnostics.

The absence of a clear “genotype-CHD” correspondence suggests a polygenic nature of the heart anomalies development. Through various methodological scientific research, new genes have been identified that show an association with the formation of cardiac anomalies [18–20], making them potentially applicable for predicting disease development. This is further supported by the presence of high-risk variants within our cohort analysis (Figure 3A). An important result was also the identification of phenotype-specific gene associations. For example, *JARID2*, part of the Polycomb repressive complex, and *PCDHA9*, a member of the cadherin family, showed significant levels of association with specific groups of CHD (Figure 3B).

The search for other variants in a small cohort is limited by low statistical power and is prone to false positives. The applied filtering (gnomAD AF < 0.1%, CADD > 20, presence > 15%) identified 59 candidates, but most (*TEX101*, GCA, TBC1D8, and others) were previously not associated with CHD. Thus, these new associations may reflect sequencing artifacts or population stratification. Validation in a larger sample is necessary.

In summary, this work provides a systematic analysis of the genetic determinants of CHD, including the identification and functional interpretation of both coding and non-coding variants. Despite working with a small patient cohort, we assessed the potential diagnostic applicability of genetic predictors of CHD proposed in the literature. We demonstrated the limited scope of existing annotation resources. Simultaneously, for some CHD-associated genes cited in the literature, we showed reproducibility of their risk potential within our cohort. It is important to note the limitations of our sample, related to low statistical power and low replication of published associations, possibly due to differences in LD structure. We also highlighted the importance of considering the specific form of CHD in genetic risk analysis, as well as the value of integrating clinical classification, genomic data, and expression profiles to identify phenotype-specific associations. Additionally, *de novo* variants and regulatory signals with potential significance for the pathogenesis of CHD were identified. The results obtained expand the understanding of the genetic factors underlying the formation of congenital heart anomalies and demonstrate the need for further research in this area, considering specific CHD phenotypes. We emphasise that the findings presented herein represent statistical associations rather than proven causal mechanisms, and all reported variants require independent validation before any clinical interpretation.

## Data Availability

The whole-genome sequencing datasets analyzed in this study are not publicly available because they contain potentially identifying genomic information, and their public deposition is restricted by the informed consent provided by participants and the approval of the institutional ethics committee. Requests to access the datasets should be directed to Dmitry Svetlichnyy, dsvetlichnyjl@cspfmba.ru.
