# Supplementary material for: Congenital heart defects genetic architecture in a small cohort: an integrated approach to prioritizing variants
Source: Front Cardiovasc Med. 2026 Jul 6;13:1850208. doi: 10.3389/fcvm.2026.1850208 (PMC13381780; doi:10.3389/fcvm.2026.1850208)
Supplement: Supplementary file 2 [file Table1.pdf]

Supplementary Material

1 SUPPLEMENTARY TABLES AND FIGURES

1.1 Tables

Table S1. Quality metrics for study cohorts (CHD (case) and control)

	Average	Min	Max	StdDev
Control	44.5	30.01	64.1	6.8
Case	42.8	31.18	56.2	5.3

Table S2. CHDdb/CHDgene annotation of genes from Figure 3 (https://chdgene.victorchang.edu.au; https://chddb.fwgenetics.org)
table available in separate supplementary file ***"Table S2"***

Table S3. CHD cohort- and subgroup-enriched SNPs (in CHD-genes from Figure 3)
table available in separate supplementary file ***"Table S3"***

Table S4. Representation of SNPs CHD associated according to data *Lahm et al., 2021* in the study sample

SNP	Groupe	Gene	Common (p value/ OR)
rs185531658	all CHD and septal defects	none	p-value: 0.2087 / OR: 0
rs149890280	TGA subgroup	MACROD2	p-value: 0.1297 / OR: 0
rs150246290	TGA subgroup	MACROD2	p-value: 0.1297 / OR: 0
rs148563140	TGA subgroup	ZBTB10	p-value: 0.7554 / OR: 0.7184
rs143638934	TGA subgroup	ZBTB10	p-value: 0.7554 / OR: 0.7184
rs149467721	TGA subgroup	MACROD2	p-value: 0.4677 / OR: 0.4757
rs77094733	TGA subgroup	MACROD2	p-value: 0.4677 / OR: 0.4757
rs146300195	TOF (right heart lesions)	SLC27A6	p-value: 0.6769 / OR: 0
rs3547121	left heart lesions	ARHGEF4	none
rs114503684	left heart lesions	TFDP2	p-value: 0.3011 / OR: 2.333
rs2046060	left heart lesions	none	p-value: 0.1375 / OR: 1.383
rs138741144	septal defects	ASIC2	p-value: 0.2714 / OR: 0.451
rs870142	ASD	STX18-AS1	p-value: 0.5056 / OR: 0.8484
rs145619574	ASD type II	WDR7	p-value: 0.5632 / OR: 1.932
rs72917381	ASD type II	WDR7	p-value: 0.5632 / OR: 1.932
rs187369228	ASD type II	LEPREL1	p-value: 0.741 / OR: 1.447
rs17677363	ATAV	GOSR2	p-value: 0.4467 / OR: 1.292
rs11874	ATAV	GOSR2	p-value: 0.4467 / OR: 1.292
rs76774446	ATAV	GOSR2	p-value: 0.4467 / OR: 1.292
rs117527287	ATAV	TBX18	p-value: 0.3502 / OR: 0

Table S5. CADD ($\geq$ 15) and DANN ($\geq$ 0.5) pathogenicity scores for SNPs enriched in the CHD cohort and subgroups (from Figure 3)

SNP	P adj	Groupe	Gene	CADD	DANN	Ensembl Regulatory Build	GeneHancer
3_38488334_C_T	0,01558	q25.0	ACVR2B	15,43	0,72000566286476		
2 _131007541 _A _G	0,02504	common	ARHGEF4	15,24	0,950523301141374		
17_33141383_G_A	0,00428	q21.0	ASIC2	15,6	0,798739738828392		
17_33179446_T_C	0,04519	q21.0	ASIC2	19,94	0,679675219890621		
17_33273281_A_G	0,02107	q25.0	ASIC2	21,6	0,615579764397369		
17_33894144_C_T	0,04513	q25.0	ASIC2	17,04	0,762311566558652		
16 _3781229 _G _T	0,04475	common	CREBBP	22,5	0,966120370377183		
16_3784315_T_C	0,04766	q21.0	CREBBP	15,32	0,531398980030253		
11 _6640369 _G _T	0,006881	common	DCHS1	22,3	0,642818845433937		
11 _6644875 _T _C	0,03953	common	DCHS1	15,34	0,989915341144048		
11_6640369_G_T	0,00323	q21.1	DCHS1	22,3	0,642818845433937		
11_6644875_T_C	0,04615	q23.4	DCHS1	15,34	0,989915341144048		
11_128526052_A_G	0,03594	q21.2	ETS1	21,6	0,782770099958914		Promoter/Enhancer
11_128568530_A_G	0,02106	q21.2	ETS1	15,01	0,530508057619587		Enhancer
11_128576018_A_G	0,04697	q21.2	ETS1	16,38	0,753716713552189		Enhancer
11_128526052_A_G	0,03108	q25.0	ETS1	21,6	0,782770099958914		Promoter/Enhancer
19_18895839_A_G	0,03407	q21.2	GDF1	18,55	0,995558062191274	promoter	Promoter/Enhancer
6 _15304642 _A _G	0,01098	common	JARID2	16,18	0,758951560937274		Enhancer
20 _14234955 _T _G	0,04618	common	MACROD2	16,45	0,684164199007609	promoter flanking region	
20 _14720911 _G _T	0,00255	common	MACROD2	15,01	0,779531650592375		
20 _15173692 _T _C	0,003274	common	MACROD2	15,97	0,705181800408348		
20 _15223247 _T _C	0,00249	common	MACROD2	17,23	0,7218855723922	promoter flanking region	
20_14545665_A_G	0,04174	q21.1	MACROD2	19,12	0,643158894928377		
20_14919158_C_A	0,04366	q21.1	MACROD2	19,62	0,937737747525889		Enhancer
20_15913475_T_A	0,0376	q21.2	MACROD2	17,88	0,792841756152574		Enhancer
20_15297868_A_G	0,03539	q23.4	MACROD2	19,41	0,935440153137467		
20_15574818_G_A	0,04231	q23.4	MACROD2	15,08	0,653941339497112		
20_14716740_A_G	0,02834	q25.0	MACROD2	17,93	0,879065842478539	promoter flanking region	Enhancer
20_14720911_G_T	0,02107	q25.0	MACROD2	15,01	0,779531650592375		
20_15046986_A_C	0,04331	q25.0	MACROD2	15,04	0,647391438302018		
20_15146953_G_A	0,04445	q25.0	MACROD2	16,18	0,700134987375313	enhancer	
5_140857365_C_G	0,0004028	q21.0	PCDHA9	22,1	0,997901616383674		
5_140857573_G_A	0,04766	q21.0	PCDHA9	23,1	0,779796464274964		
5_140925349_A_G	0,04825	q21.0	PCDHA9	18,51	0,549175362172374		
4 _26321460 _G _C	0,04645	common	RBPJ	18,24	0,887184233522742	promoter	Promoter/Enhancer
4_26321460_G_C	0,04073	q23.4	RBPJ	18,24	0,887184233522742	promoter	Promoter/Enhancer
7_83448186_C_T	0,03006	q21.0	SEMA3E	16,69	0,589330571654615		
7_83427810_T_C	0,005677	q23.4	SEMA3E	15,41	0,629239511119963	promoter flanking region	Enhancer
7_83546946_A_G	0,01388	q25.0	SEMA3E	15,39	0,787171644211234	enhancer	
5_128538228_C_G	0,01685	q21.2	SLC27A6	15,15	0,72275576005496	promoter	Promoter/Enhancer
5_128994400_C_T	0,02055	q23.4	SLC27A6	15,76	0,996879142202353		
22_19765420_A_C	0,01391	q21.0	TBX1	18,79	0,689722709521635		Enhancer
7_35253582_A_G	0,0007582	q25.0	TBX20	15,6	0,778253148927688	promoter	Promoter/Enhancer
18 _56984239 _C _T	0,003668	common	WDR7	19,04	0,683948538863394		
18 _56984910 _G _A	0,01959	common	WDR7	16,85	0,740262128072824	open chromatin region	
18_56785228_C_T	0,04697	q21.2	WDR7	15,4	0,870717767076311	open chromatin region	
18_57030691_G_A	0,04667	q23.4	WDR7	16,9	0,697545785004228	open chromatin region	Enhancer
8 _105491617 _A _G	0,00663	common	ZFPM2	16,83	0,503829491785646		
8_105747488_A_G	0,02397	q21.0	ZFPM2	21,1	0,528979650463205	promoter flanking region	Enhancer
8_105688059_G_A	0,04071	q21.2	ZFPM2	17,81	0,768054179433049		
8_105784754_C_T	0,03094	q21.2	ZFPM2	15,79	0,618123781375163		

Table S6. CHD cohort- and subgroup-enriched SNPs (in not-CHD-genes from Figure 4)
*table available in separate supplementary file **"Table S6"***

Table S7. CADD ($\geq$ 15) and DANN ($\geq$ 0.9) pathogenicity scores for SNPs enriched in the CHD cohort (from Figure 4)

Chr	Position	Ref	Alt	Rs ID	Type	Gene	CADD	DANN	eQTL (4F)	Regulome
chr19	984537	A	G	rs61732720	Coding	WDR18	24,1	0,998277612571757	-	-
chr6	33173503	G	A	rs2855430	Coding	COL11A2	33	0,994968313113851	-	-
chr4	177335759	C	G	rs7689099	Coding	NEIL3	21,7	0,942022896250334	-	-
chr3	142823607	G	T	rs17554211	Coding	PCOLCE2	23,1	0,927690843272833	-	-
chr14	54981691	G	C	rs61741224	Coding	WDHD1	24,9	0,92029579305567	-	-
chr5	169308410	A	C	rs56788493	Non-coding	-	21,6	0,995645445444895	-	-
chr6	33272092	C	T	rs17215231	Non-coding	-	21,7	0,99348874373566	eQTL	1f
chr6	108985478	T	C	rs12212402	Non-coding	-	20,7	0,976740564131554	-	1f
chr3	132172193	A	G	rs73211906	Non-coding	-	21,8	0,972885518736188	-	1f
chr10	8910589	C	T	rs17413266	Non-coding	-	20,5	0,967232783150895	-	2b
chr18	41028818	A	G	rs16973990	Non-coding	-	21,5	0,961782594367386	-	5
chr16	17147499	T	C	rs2125192	Non-coding	-	21,9	0,948791364690142	-	6
chr20	31587870	T	G	rs113882388	Non-coding	-	20,6	0,930921212833124	-	1f
chr9	2018965	T	C	rs72687570	Non-coding	-	20,4	0,929624616177717	-	2a
chr13	94061141	T	C	rs17791653	Non-coding	-	21,2	0,913896463602244	-	4
chr7	128106341	C	A	rs57463770	Non-coding	-	21,5	0,901117911613595	-	1b

Table S8. GTEx eQTL SNP (Figure 4F)

Gene	Rs ID (dbSNP155 GRCh38p13)	Slope
PTCH1	rs145965565	0.233005
B3GALT4	rs17215231	0.204561
RPS18	rs17215231	-0.771197
RPS18	rs17215231	-0.809087
B3GALT4	rs17215231	0.379923
RPS18	rs17215231	-0.842232
B3GALT4	rs17215231	0.362544
RPS18	rs17215231	-0.811688
FCRL6	rs3027034	-0.31226
GPR27	rs73090632	0.870454
GPR27	rs73090632	0.659976
EIF4E3	rs73090632	0.180956
GPR27	rs73090632	0.607838

1.2 Figures

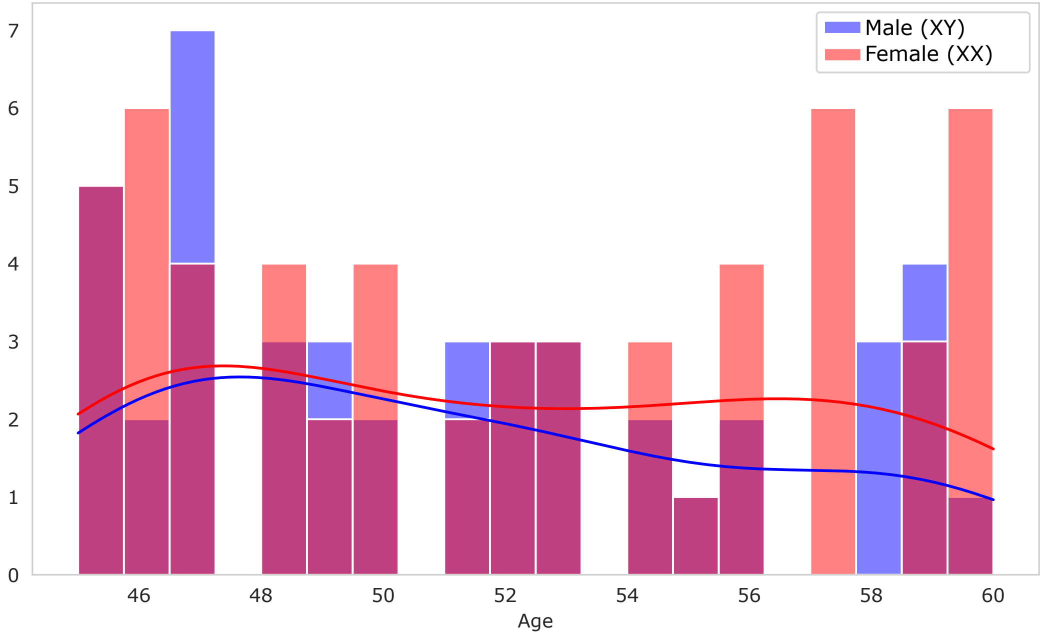

Figure S1. Control group age and sex distribution

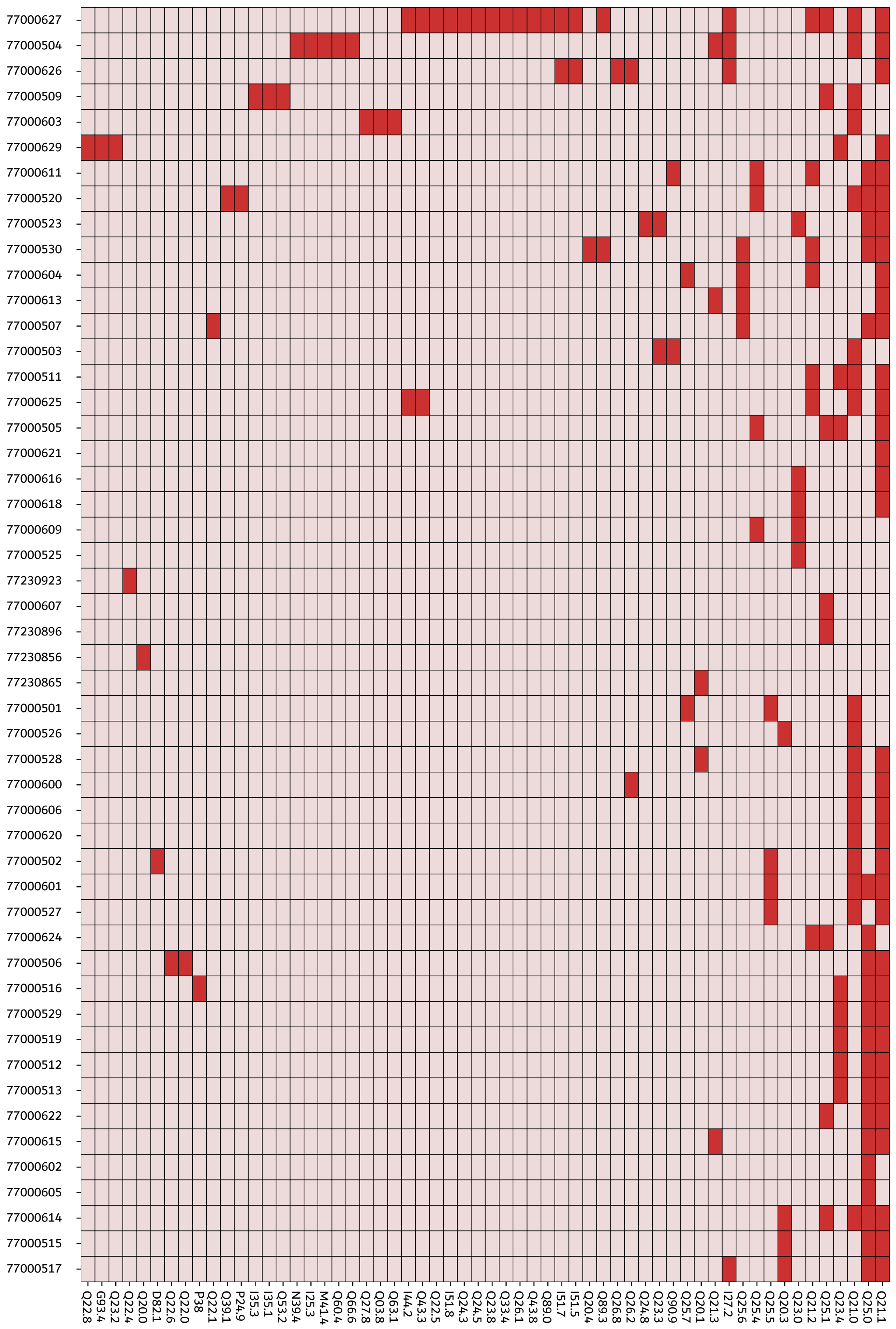

Figure S2. Representation of CHDs within the study sample

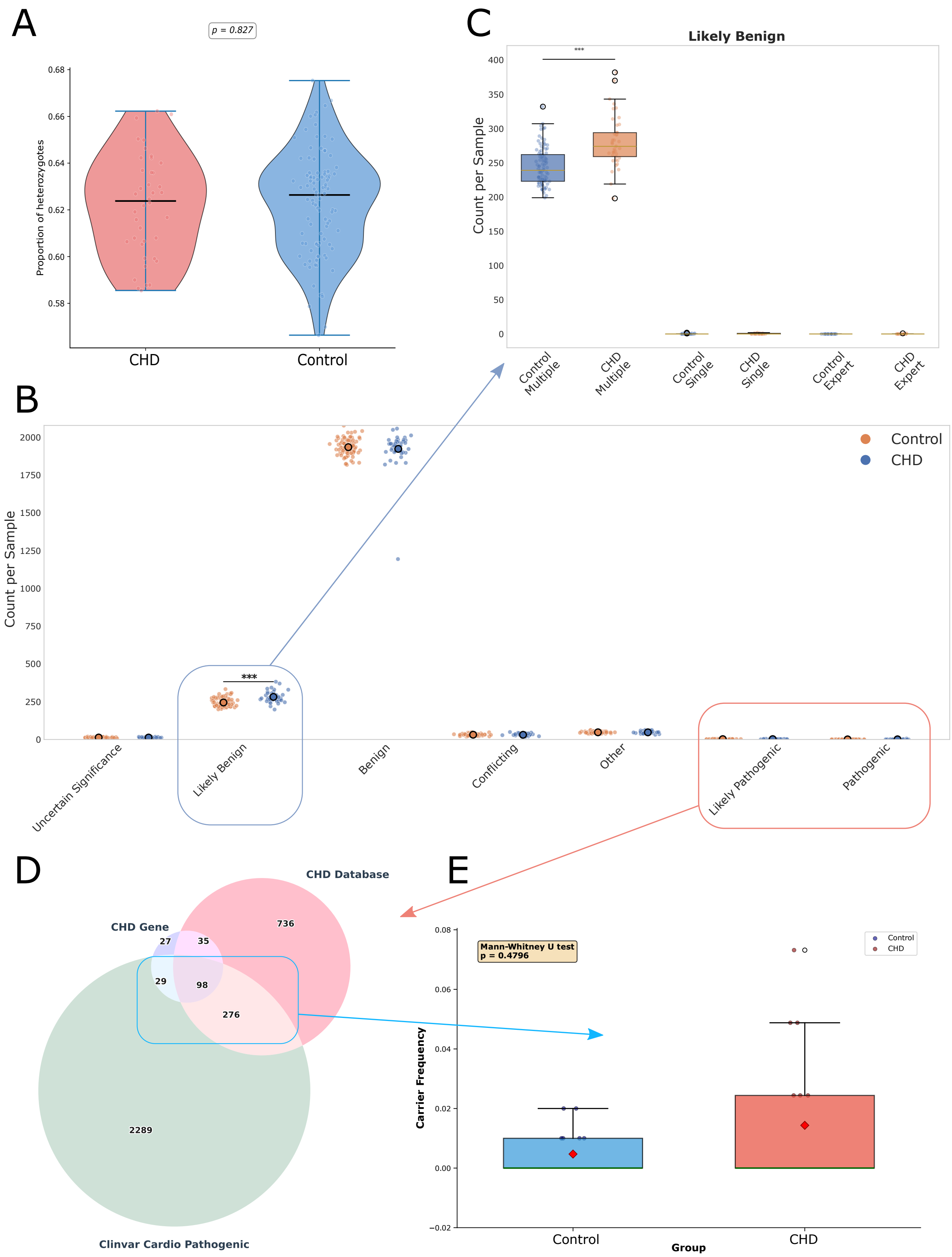

Figure S3. Cardiac SNP representation in study cohorts.
A Heterozygote proportion by cohort. **B** Functional annotation stratification of cardiac ClinVar SNPs. **C** ClinVar submitter-tier analysis of study variants. **D** Overlap with public CHD database variants (https://chdgene.victorchang.edu.au; https://chddb.fwgenetics.org). **E** Representation of dual-annotated cardiac SNPs (ClinVar + public CHD databases).

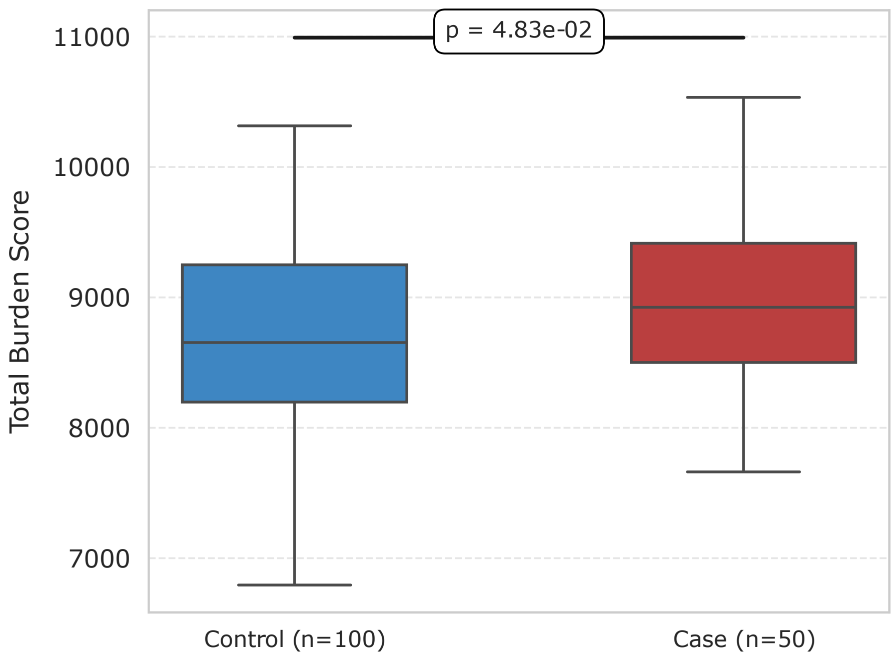

Figure S4. Plot of mutation burden distribution in patients compared to the control sequence (Mann-Whitney U test)

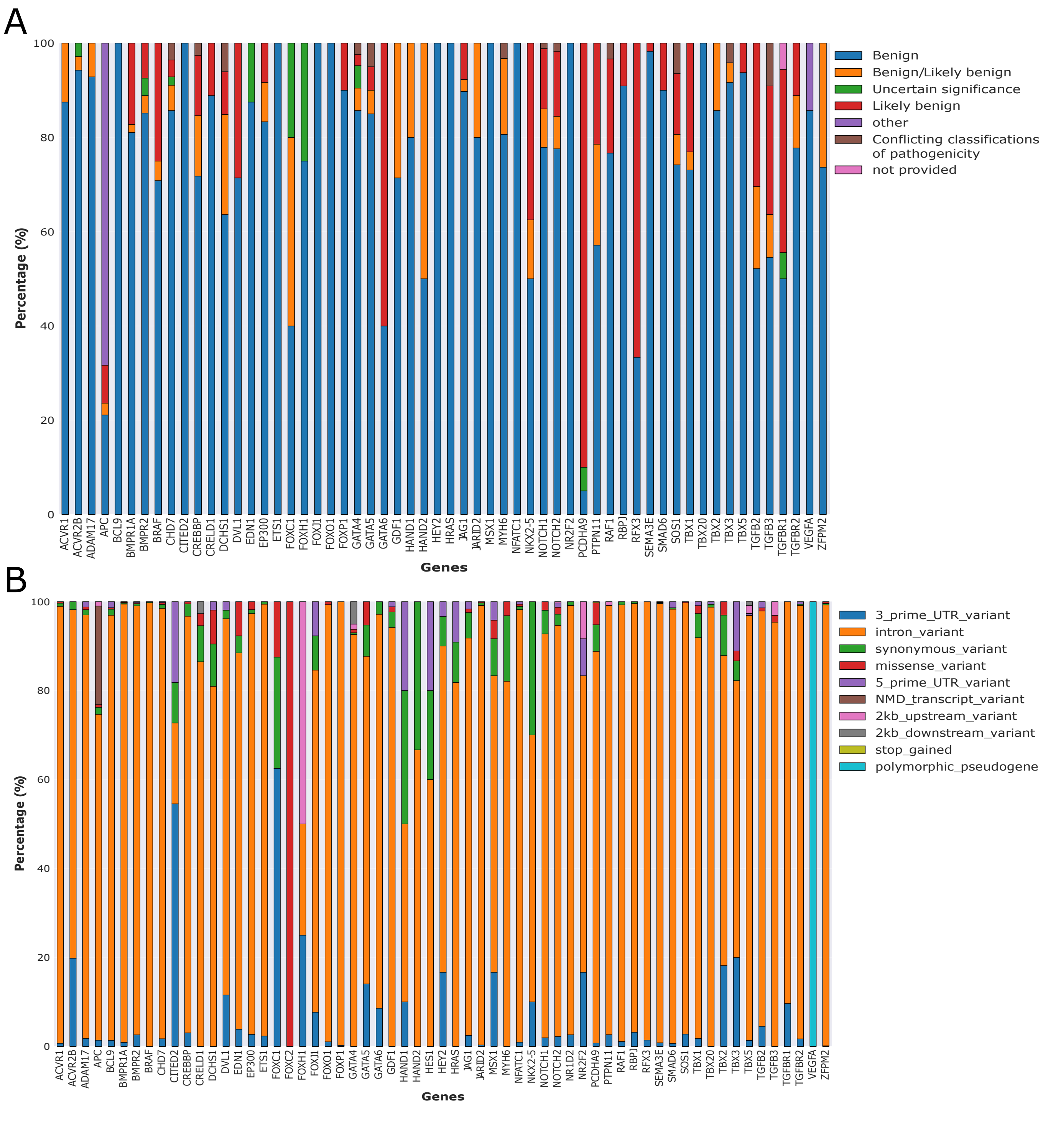

Figure S5. Annotation of SNPs in CHD-associated genes.
A ClinVar clinical classification of identified variants **B** Functional annotation of SNPs by genomic context

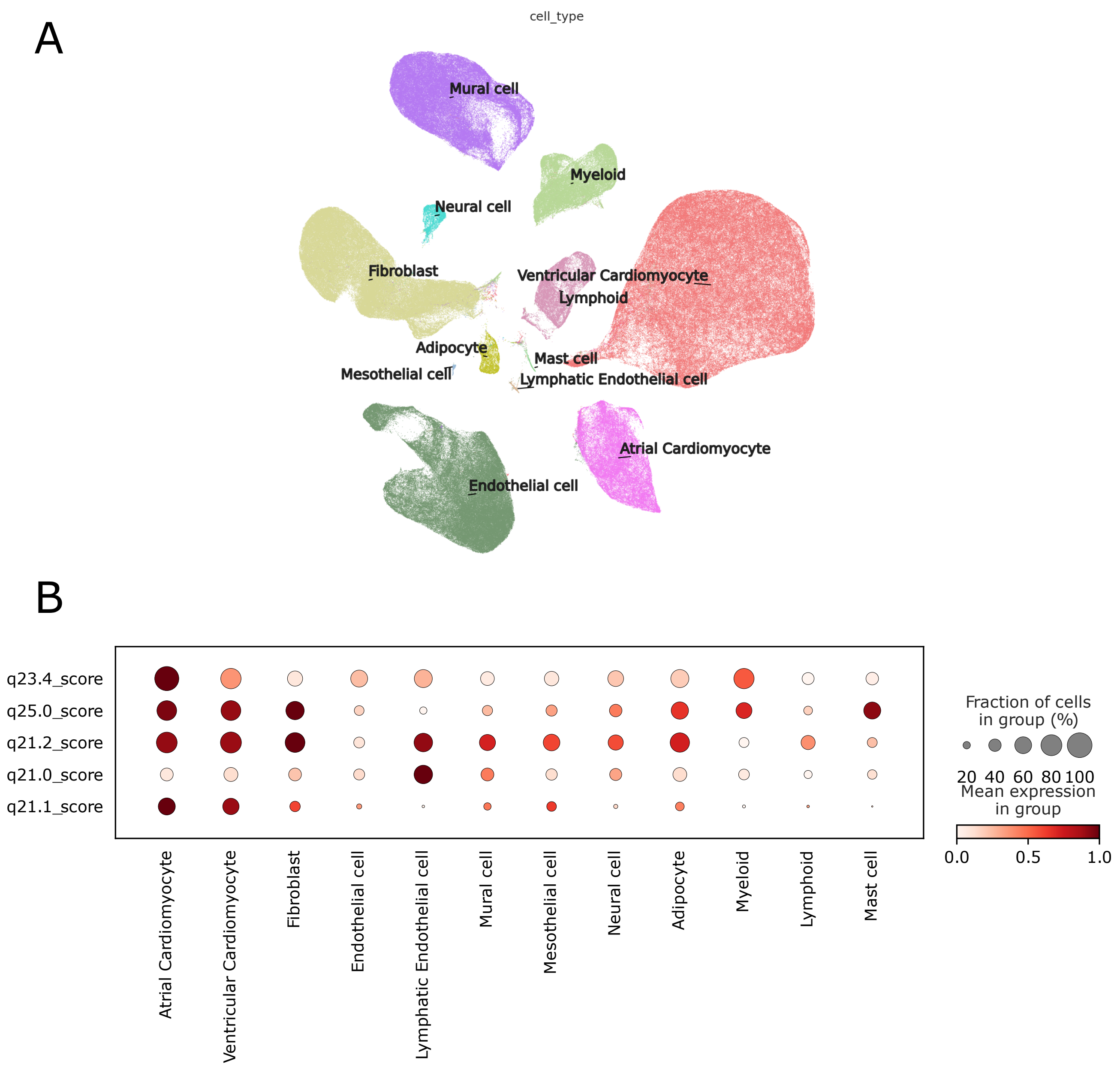

Figure S6. Modeling expression in functional categories of genes associated with CND.
A UMAP projection of single-cell sequencing of the cardiac atlas (https://www.heartcellatlas.org/). **B** Assessment of the expression module in cell types of the cardiac atlas (genes were selected as the most distinctive and represented in the chosen nomenclature, according to 3B).
